# Strain differences in the response of rats to the injection of nickel sulphide.

**DOI:** 10.1038/bjc.1966.102

**Published:** 1966-12

**Authors:** M. R. Daniel

## Abstract

**Images:**


### STRAIN DIFFERENCES IN THE RESPONSE OF RATS TO THE INJECTION OF NICKEL SULPHIDE

MARY R. DANIEL

*From the Division of Histology, Ontario Veterinary College, Guelph, Canada*

*Present address: Strangeways Research Laboratory, Cambridge, England*

IT has been shown by Gilman and Ruckerbauer (1962) and Gilman (1962) that the intramuscular injection of nickel sulphide into either Hooded or Fischer rats produces a high incidence of tumours, many of which are well differentiated rhabdomyosarcomata. Thus of two groups of Hooded rats receiving injections of refinery dust with a nickel sulphide ($Ni_3S_2$) content of 57·0%, 70–71% developed tumours with an average latent period of 160–170 days; and of Fischer rats with injected nickel sulphide, 89% developed tumours within an average of 150 days.

By contrast, Payne (1964) found that Bethesda Black rats were relatively resistant to the carcinogenic action of intramuscularly implanted nickel sulphide. The final proportion of animals that developed tumours was high (74%), but the latent period was longer than for the Hooded and Fischer rats examined by Gilman; the first tumours were produced after 15 months, the average latent period being 17·3 months. These tumours were described as rapidly growing, anaplastic sarcomata.

As part of an investigation into the cause of this difference in response, a study has been made of the short- and long-term reactions of the muscle of the three strains—Hooded, Fischer and Bethesda Black—to powdered nickel sulphide. The results obtained indicated that the relative resistance of the Bethesda Black rats may be related to the intense and prolonged phagocytic response to the powdered carcinogen which was observed in this strain.

### MATERIALS AND METHODS

Male and female rats, of the closed colonies of Hooded, Fischer and Bethesda Black strains of the Department of Histology, Ontario Veterinary College, were used. The animals were approximately 2–3 months old at the start of the experiments.

One tenth of a ml. of a suspension of nickel sulphide ($Ni_3S_2$) in aqueous penicillin G procaine ("Ayercillin"), containing 100 mg. $Ni_3S_2$/ml., was injected intramuscularly into each gastrocnemius of every rat.

The first experiment was designed to assess the relative sensitivities of Hooded and Bethesda Black rats to the carcinogenic action of the nickel compound, under the experimental conditions used. Fifteen male and 15 female Hooded, and 15 male and 12 female Bethesda Black rats were used. Those animals which developed tumours during the experiment were killed for autopsy when visible tumours appeared. One sample of each sex of the Bethesda Black rats, without apparent tumours, was killed for examination $4\frac{1}{2}$ and 10 months after injection, and the rats remaining after 14 months were autopsied whether or not they had developed tumours.

In the second experiment, a comparison was made of the short-term response of the muscle tissue of the three strains to the injected nickel sulphide. Five males and 10 females of each of the Hooded and Bethesda Black strains, and 10 males and 10 females of the Fischer strain, were used. Samples of both males and females were killed for examination at fortnightly intervals after the injection of the suspension.

Tissues for histological examination were fixed in Zenker-acetic, embedded in paraffin wax, and sectioned at 8 $\mu$. The sections were stained with carmalum-aniline blue-orange G.

### RESULTS

*First experiment*

The Bethesda Black rats were less susceptible than the Hooded to the carcinogenic action of the nickel sulphide. As shown in Table I, tumours were produced in all the Hooded, but in only 14 of the 27 Bethesda Black rats. Further, although almost all the Hooded rats developed tumours at the site of injection in both legs, in the Bethesda Black only one leg was affected, and the average latent period was approximately twice as long.

Although in both strains most of the tumours contained rhabdomyosarcomatous elements, in the Bethesda Black these were usually poorly differentiated, and mixed with fibro-sarcomata, reticulum cell sarcomata, or both. Some of the tumours in both strains contained phagocytes, with ingested nickel sulphide, scattered among the abnormal muscle cells.

In the 2 Bethesda Black rats examined $4\frac{1}{2}$ months after injection, and showing no sign of tumour formation, the nickel sulphide was contained in a grey soft tissue mass lying on the surface of the leg muscles ; the sulphide could not be separated from the tissue. Nickel sulphide was still present in the tissue of tumour-free Bethesda Black rats killed up to 14 months after injection. The soft tissue masses (Fig. 1) containing the sulphide were smaller than at $4\frac{1}{2}$ months, and were often scattered as small patches in the connective tissue overlying the muscle, which appeared normal to the naked eye.

Histological examination of these masses showed that most of the nickel was within phagocytes (Fig. 2). These were sometimes embedded in a loose network of thick connective tissue bundles, and, in the rats killed after 14 months' exposure to the sulphide, were often associated with large numbers of lymphocytes (Fig. 2) ; in approximately half the animals, variable numbers of plasma cells were also seen at the peripheries of the phagocyte aggregates.

In a few of the Bethesda Black rats exposed to nickel sulphide for 14 months, the powder was also present as masses, with associated necrotic tissue, in spaces lined with foreign body giant cells. In one rat there was a region of early fibro-sarcoma within the phagocyte mass.

The muscle fibres nearest to the phagocyte aggregates, from which they were usually separated by variable amounts of fairly dense connective tissue, were somewhat narrower than the mature muscle further away, and many of them showed abnormalities in the direction of their fibrils ; some were " ring fibres " (Fig. 3) and in others the fibrils formed interlacing networks (Fig. 4). Similar

TABLE I.—*Tumour Production in Hooded and Bethesda Black Rats in Response to Intramuscular Injection of Nickel Sulphide*

| Strain | Sex | Proportion of rats with tumours | Number of tumours | Period for development of visible tumour (weeks) | Number of tumours containing | | | | | | |
|---|---|---|---|---|---|---|---|---|---|---|---|
| | | | | | Rhabdomyosarcoma | | | | Fibrosarcoma | Reticulum cell sarcoma | Other components |
| | | | | | [2] Poor | Fair | Good | Very good | | | |
| Hooded | ♂ | 15/15 | 28 | 17–26 | 4 | 6 | 17 | 0 | 4 | 1 | 0 |
| | ♀ | 13/13 | 26 | 16–22 | 2 | 7 | 15 | 2 | 1 | 2 | 1 (Haemangio-sarcoma) |
| Bethesda Black | ♂ | 7/13[1] | 7 | 23–57 | 4 | 2 | 0 | 1 | 3 | 4 | 1 (Lympho-sarcoma) |
| | ♀ | 7/10 | 7 | 19–56 | 1 | 4 | 0 | 0 | 3 | 3 | 0 |

[1] The total numbers of male and female Bethesda Black rats are those at the conclusion of the experiment (14 months after injection).

2 rats of each sex, killed at 4½ and 10 months after injection, were tumour-free.

[2] Degree of differentiation.

appearances were seen in groups of fibres, or individual fibres, at some distance from the deposit of nickel sulphide. A few fibres showing Zenker's degeneration were also present.

In the Hooded and Bethesda Black rats in which tumours were present, and from which normal muscle adjacent to the tumours was examined histologically, no ring fibres were seen in this tissue.

In approximately half of the Bethesda Black rats killed 14 months after injection, the popliteal, inguinal or lumbar lymph nodes contained macroscopically visible deposits of nickel sulphide ; histologically, these were seen to consist of phagocytes with ingested sulphide. Some lymph nodes also contained aggregates of similar cells devoid of the powder (Fig. 5). Deposits of sulphide were not seen in the lymph nodes of tumour-bearing rats of either strain.

In the 2 Hooded rats which developed tumours in one leg only, no sulphide could be seen at the injection site in the unaffected leg.

*Second experiment*

The main results are summarized in Table II.

TABLE II

| Histological findings | Strain | Time in weeks | | | | |
|---|---|---|---|---|---|---|
| | | 2 | 4 | 6 | 8 | 10 |
| $Ni_3S_2$ loose in muscle | Fischer | + | — | — | — | — |
| | Hooded | + | — | — | — | — |
| | Bethesda Black | + | — | — | — | — |
| $Ni_3S_2$ in sac with necrotic tissue | Fischer | + | +++ | +++ | ++ | ++ |
| | Hooded | + | +++ | ++ | ++ | (+) |
| | Bethesda Black | + | +++ | (+) | — | — |
| $Ni_3S_2$ in phagocytes at injection site | Fischer | — | (+) | (+) | + | + |
| | Hooded | — | (+) | + | + | + |
| | Bethesda Black | — | + | ++ | +++ | +++ |
| $Ni_3S_2$ in phagocytes in lymph nodes | Fischer | — | (+) | + | (+) | (+) |
| | Hooded | — | + | ++ | + | + |
| | Bethesda Black | — | + | +++ | +++ | ++ |
| Abnormal myoblasts and muscle straps present | Fischer | — | — | + | + | ++ |
| | Hooded | — | — | — | + | +++ |
| | Bethesda Black | — | — | — | — | — |

Two weeks after the injection of powdered nickel sulphide, the injection site appeared macroscopically similar in the three strains. The powder was partially localized in one or more sacs within the muscle tissue, and between the tissue planes, although some was still loose on the surface of the muscle. No sulphide could be seen with the naked eye in the regional lymph nodes.

Histological examination of the site showed that all the tissue immediately in contact with the nickel sulphide was dead. In all three strains, there was a surrounding zone where little or no particulate sulphide could be seen, but many muscle fibres were necrotic and were replaced by a collection of cells forming the " muscle cell tubes " of Waldeyer (Fig. 6). In some of the necrotic fibres, the peripheral cytoplasm appeared healthy, and had formed multinucleated masses and straps (Fig. 7). In the Hooded and Fischer rats, in contrast to the Bethesda Black, there was much muscle regeneration in this zone ; many myoblasts, some in mitosis, and muscle straps were seen.

After 4 weeks' exposure to nickel sulphide, the three strains showed a localization of the powder in one or more soft tissue masses, approximately 5 $\times$ 2 mm., usually lying between the heads of the gastrocnemius or on the surface of this muscle. In many of these masses, the powder lay in the centre of a sac containing necrotic tissue ; the wall of the sac apparently contained nickel sulphide inextricably bound up with the tissue. Some sulphide was present in the local and lumbar lymph nodes of the Hooded and Bethesda Black rats, and a small amount was seen in the lumbar nodes of the Fischer rats.

Histologically, the tissue immediately adjacent to the deposits of nickel sulphide was necrotic. In the Hooded rats, this region was surrounded by a zone of intense regenerative activity, with accompanying degeneration ; multinucleated masses and muscle straps could be seen, and there were a few mitotic figures. In one of these rats, some granulation tissue, with phagocytes containing nickel sulphide, was also present.

In the Bethesda Black rats, the granulation tissue surrounding the necrotic zone was more abundant, and consisted of a loose network of dense connective tissue bands containing pockets of phagocytes laden with nickel sulphide. The granulation tissue was bordered by a thin band of immature muscle fibres and

---

### EXPLANATION OF PLATES

Fig. 1.—Bethesda Black rat, 57 weeks after injection. Site of implantation. Phagocyte aggregates in loose connective tissue. $\times$37.

Fig. 2.—Part of one aggregate shown in Fig. 1. Granules of nickel sulphide are seen in pale phagocytes. Many lymphocytes and some plasma cells are also present. $\times$460.

Fig. 3.—Bethesda Black rat, 57 weeks after injection. Muscle near nickel sulphide deposit. Ring fibres are present ; the annular portions of most of these show cross striations. $\times$460.

Fig. 4.—Bethesda Black rat, 57 weeks after injection. Muscle near nickel sulphide deposit. Aberrant fibril bundles in a muscle fibre. $\times$760.

Fig. 5.—Bethesda Black rat, 57 weeks after injection. Lymph node. Aggregates of large pale-staining cells, devoid of nickel sulphide. $\times$460.

Fig. 6.—Bethesda Black rat, 2 weeks after injection. Muscle near implantation site. The fibres are degenerating, with the formation of " muscle cell tubes ". $\times$460.

Fig. 7.—Fischer rat, 2 weeks after injection. Muscle near injection site. The central parts of the fibres have broken down, and the surviving peripheral regions have formed multinucleated masses and straps. $\times$460.

Fig. 8.—Bethesda Black rat, 8 weeks after injection. Mature muscle near implantation site. One fibre shows longitudinal splitting into component fibril bundles. $\times$460.

Fig. 9.—Bethesda Black rat, 6 weeks after injection. Site of implantation. Almost all the sulphide is associated with large, pale-staining cells, most of which look healthy. $\times$690.

Fig. 10.—Hooded rat, 6 weeks after injection. Site of implantation. A few phagocytic cells are present. Some nickel sulphide is associated with these cells, many of which are degenerating. Free nickel sulphide is also present. $\times$690.

Fig. 11.—Fischer rat, 6 weeks after injection. Site of implantation. Many extravasated erythrocytes are present, and much of the nickel sulphide is free. Many of the cells are degenerating or dead. $\times$690.

Fig. 12.—Bethesda Black rat, 10 weeks after injection. Lymph node. A focus of sulphide-laden phagocytes, some of which are degenerating. $\times$460.

Fig. 13.—Bethesda Black rat, 10 weeks after injection. Site of implantation. Most of the nickel sulphide is within pale-staining phagocytic cells, enmeshed in connective tissue. Bands of lymphocytes are present at the periphery of the aggregate of phagocytes. $\times$460.

Fig. 14.—Hooded rat, 10 weeks after injection. Site of implantation. Abnormal, probably malignant muscle straps in dense connective tissue, with a few sulphide-containing phagocytes. $\times$460.

Fig. 15.—Fischer rat, 10 weeks after injection. Site of implantation. Abnormal myoblasts in dense connective tissue. Nickel sulphide is present, both free and associated with cells. $\times$460.

Daniel.

Daniel.

Daniel.

Daniel.

muscle straps, scattered among which were a few phagocytes containing sulphide or yellow-brown pigment.

In the Fischer rats, the necrotic region containing the nickel sulphide was surrounded either by granulation tissue containing some phagocytic cells or by regenerating muscle, including muscle straps and immature striated fibres. Some mitoses were seen in this tissue.

In all three strains, the mature muscle surrounding the reaction zone showed histological evidence of sublethal damage, including sub-sarcolemmal " rowing " of nuclei and a longitudinal splitting of fibres into smaller bundles of fibrils. These features persisted throughout the early stages of the response to the injection (Fig. 8).

The nickel sulphide in the lymph nodes was contained within groups of phagocytes.

In one of the male Fischer rats killed at this stage, the kidneys had rough surfaces and were found on histological examination to contain regions of proximal convoluted tubular necrosis.

By the sixth week after injection there were macroscopic differences in the tissue response at the site, between the Bethesda Black and the other two strains. In the Hooded and Fischer rats, the nickel sulphide lay in a sac between the heads of the gastrocnemius, and could be scraped out as a loose paste mixed with necrotic tissue ; some sulphide was also mixed with the tissue of the wall of the sac. By contrast, in 2 of the 3 Bethesda Black rats the nickel sulphide could not be removed from the tissue in which it was localized and to which it gave a grey colour. The bands of mixed tissue and sulphide lay in loose connective tissue on the surface of the muscle, or between its heads.

At this stage, nickel sulphide could be seen in the popliteal and lumbar lymph nodes of the three strains, but the amount was greatest in the Bethesda Black rats.

Histologically, the sulphide at the injection site in 2 of the Bethesda Black rats was contained within phagocytic cells, which formed a large mass subdivided into smaller aggregates by vascular connective tissue (Fig. 9) ; some plasma cells and a few lymphocytes were seen at the periphery of the mass of phagocytes. In one rat, in which some loose sulphide was also present, this was enclosed in a cavity lined by multinucleated giant cells. The phagocyte aggregates contained variable amounts of connective tissue, and were usually clearly demarcated from the surrounding muscle. The muscle fibres closest to the reaction zone were immature, and the mature muscle further away showed signs of sublethal damage.

In the Bethesda Black rat in which the sulphide had been contained in a sac, and in one Hooded rat, the cellular response to the nickel sulphide was qualitatively similar but quantitatively less than in most of the Bethesda Black rats ; much of the sulphide was still free, either as individual particles lying among the cells or as clumps surrounded by necrotic tissue (Fig. 10). In these and in the other Hooded rats, some haemorrhagic, vascular granulation tissue was present ; this contained some muscle straps and myoblasts, and was surrounded by a zone of muscle regeneration with some mitosis. Some degenerating muscle was also seen in the reaction zone, which was not clearly demarcated from the surrounding mature muscle.

In the Fischer rats, there was still less of an active response to the carcinogen, which was surrounded by necrotic tissue and a cellular, haemorrhagic exudate (Fig. 1). The regenerating muscle bordering this zone was not sharply defined from it, and some of the muscle fibres formed were abnormal, having an irregular shape and large nuclei.

As in the Bethesda Black rats, the mature muscle at some distance from the injection site in the Hooded and Fischer rats showed " rowing " of nuclei and longitudinal splitting. The differences in the amounts of nickel sulphide seen macroscopically in the lymph nodes were related to the size of the aggregates of sulphide-laden phagocytes seen microscopically.

The tissue response of the Bethesda Black rats killed 8 weeks after injection differed little, either macroscopically or histologically, from that seen in most of the animals of this strain killed at 6 weeks. In the Hooded and Fischer rats the macroscopic response was the same as at 6 weeks. Histologically, there was an increase in the amount of dense connective tissue surrounding the nickel sulphide deposit in the Hooded rats, and a slight increase in the cellular response in the Fischer. In both these strains, phagocytes containing nickel sulphide were intermingled with regenerating muscle, of which a number of straps appeared abnormal, with variable diameter and nuclear size.

At this stage, the popliteal and lumbar lymph nodes of the Bethesda Black and Hooded rats contained nickel sulphide, but there was relatively little in the lymph nodes of the Fischer rats. The histological changes in the nodes were as at earlier stages, but some of the phagocytes were necrotic.

The kidneys of both the male Fischer rats killed at this stage had rough surfaces, and showed histological evidence of proximal tubular necrosis. In the other animals, the kidneys appeared normal both macroscopically and microscopically.

By the tenth week, there appeared to be less nickel sulphide in the draining lymph nodes in all the strains (Fig. 12). In the Bethesda Black rats, the appearance of the injection site was unchanged (Fig. 13), except that one animal showed the first unequivocal " ring fibre " seen in the series ; this was among the mature muscle close to the mass of phagocytes. In other rats of this strain, some of the regenerating muscle formed an interweaving pattern ; some regenerating fibres were seen between neighbouring phagocyte aggregates.

In the Hooded rats killed at this stage some loose nickel sulphide still remained within the localizing mass, but an increased proportion was intimately mixed with apparently healthy tissue. Histological examination showed that, surrounding a small region of necrosis and loose sulphide, there was a region of dense, often vascular connective tissue containing various cell types, including phagocytes laden with nickel sulphide, and abnormal regenerating muscle tissue. In many places, the appearance of the muscle straps suggested the beginning of tumour formation (Fig. 14).

In the Fischer rats killed at this time, most of the nickel sulphide was still loose in thin-walled sacs between the heads of the gastrocnemius or between this and other muscles. Histologically, some necrotic material was still present around the carcinogen; some mature but necrotic muscle fibres were recognizable in this zone. The surrounding tissue consisted of variable amounts of dense, sometimes vascular connective tissue containing a mixture of cells including phagocytes with engulfed nickel sulphide, fibroblasts and myoblasts and muscle straps, many of which appeared abnormal (Fig. 15). More phagocytes were present than in the Fischer rats exposed for shorter periods to the carcinogen, but they were still much fewer than in the Bethesda Black rats from 6 weeks onward.

The male Fischer rats killed at this stage again showed, in contrast to the other animals, a patchy necrosis of the proximal convoluted tubules of the kidneys.

### DISCUSSION

The first of the experiments reported here confirms the relative resistance of the Bethesda Black strain of rats to the carcinogenic action of powdered nickel sulphide, although the latent period before the development of a tumour in a rat of this strain was shorter than in Payne's (1964) experiments. Since the experiment was ended after 14 months, it is not possible to say what the final tumour incidence would have been had these rats been exposed to the carcinogen for the same period as those in Payne's study.

The most striking difference among the three strains of rats in the early response to the injection of powdered nickel sulphide was the massive phagocytic invasion of the site in the resistant strain ; these phagocytes ingested the carcinogen without apparent damage. In the Hooded rats, some cellular reaction was seen from the sixth week, but it was much less than in the Bethesda Black rats, and the phagocytes sometimes appeared to be killed by the ingested sulphide ; much of the powder was still free, either in a mass surrounded by necrotic and reactive tissue, or among the reactive tissue cells. In the Fischer rats the phagocytic response was still less, and was delayed until a time when abnormal regenerating muscle straps were present.

It is not clear what proportion of the phagocytes arose from the " muscle cell tubes " seen in the tissue within 2 weeks of the injection of the nickel sulphide, and what proportion had invaded the tissue from elsewhere. It seems probable (Field, 1960) that some at least of the phagocytic cells in such tubes are derived from muscle tissue. In the Bethesda Black rats, in the early stages, the formation of muscle straps played less part in the repair process than in the other two strains, and it is possible that in this strain the cells of the tubes differentiated into phagocytes rather than muscle cells. Some mitoses were seen in the mass of phagocytes, but were too few to account for the great number of cells present ; it seems probable, therefore, that these cells arose from both intra- and extra-muscular sources.

The localization of the nickel sulphide within the phagocytes would probably reduce the concentration of nickel compounds available for action on the regenerating muscle at the periphery of the phagocyte aggregates, both as a direct result of intracellular localization and through transportation of the powder to the regional lymph nodes. At all stages of the early reaction, the lymph nodes of the Bethesda Black rats contained larger accumulations of sulphide-laden phagocytes than did those of either of the other strains. A further observation in support of this suggestion is that in the Fischer rats, in which the phagocytic response was least, continuing muscle necrosis could be seen as late as the tenth week after injection. The renal tubular damage seen in some of the male Fischer rats could possibly have been a toxic necrosis, due to the absorption of relatively more soluble nickel from the site in this strain.

The mode of formation of the muscle fibres with aberrant fibrils, in the Bethesda Black rats exposed for periods of 10 weeks and more to nickel sulphide, is not clear. The histological evidence indicates that they might have arisen either from the intertwining developing fibres seen in some areas of muscle regeneration or from mature muscle which had been only partially damaged. A feature of the mature muscle surrounding the phagocyte masses in the early stages of the reaction to nickel sulphide was the number of fibres showing " rowing " of nuclei, a phenomenon which, according to Mason (1960), may represent a sublethal reaction to injury. There were also areas in which muscle fibres appeared to have been split longitudinally into component bundles of fibrils. Berthrong and Griffith (1961) have suggested that formation of ring fibres may result from the occurrence of muscle regeneration in the absence of a sarcolemma ; it is possible that, in this way, the ring fibres arose from an aberrant development of the peripheral region of the fibres showing nuclear rowing, while fibres with interweaving fibrils resulted from a reconstitution from the fibril bundles of a split fibre.

In those Bethesda Black rats which developed tumours, most of these were rhabdomyosarcomata, as in the Hooded rats in the present investigation and in the Hooded and Fischer rats studied by Gilman (1962). They were, however, less well differentiated than those in the Hooded rats, and were often mixed with fibrosarcomata and reticulum cell sarcomata. In Payne's experiments (1964), the Bethesda Black rats developed tumours which were described as " rapidly growing, anaplastic sarcomata " ; it is possible that these, and the mixed tumours produced in the present experiments, were derived from the phagocytic tissue formed in response to the nickel sulphide in this strain. In the present series, an early fibrosarcoma was seen in the centre of a phagocyte mass in one of the Bethesda Black rats exposed for 14 months to the carcinogen.

Two interesting features of these phagocyte aggregates were their persistence for at least 14 months and their frequent association with plasma cells and, particularly in the later stage, with large numbers of lymphocytes. There is an increasing amount of evidence (e.g. Fagraeus, 1948 ; Halpern, 1959 ; Fishman, 1961 ; Harris, 1966) that the reaction of an antigen with macrophages is necessary for the subsequent induction of antibody production by lymphocytes. It is possible that the nickel sulphide combines either with necrotic muscle protein before ingestion by the phagocytes, or with intracellular material in these cells, to form foreign proteins which are then able to induce antibody formation by the surrounding lymphocytes. The existence of such an antibody would be expected to reduce the availability of nickel compounds in a form suitable for action on muscle, and also, if it were an antibody to a nickel-muscle protein complex, to cause the destruction of muscle fibres which subsequently took up sublethal quantities of nickel.

### SUMMARY

The responses of rats of the Hooded, Fischer and Bethesda Black strains to intramuscular implants of powdered nickel sulphide have been compared. In confirmation of Payne's results, it has been found that the Bethesda Black strain is more resistant than the Hooded to the carcinogenic action of this compound.

A distinctive feature of the response of the Bethesda Black rats to the nickel sulphide was the development at the site of injection of large masses of phagocytic cells, which engulfed the powder without undergoing obvious damage. These phagocyte aggregates persisted for at least 14 months, and some were surrounded in the later stages by lymphocytes. Some of the muscle fibres near the phagocytes showed great variability in the orientation of their fibrils.

It is suggested that the ingestion of the nickel sulphide by the phagocytes may reduce the concentration of nickel compounds available for action on the muscle tissue to a subcarcinogenic level, both by the intracellular localization of the compound and by its transport to the regional lymph nodes. The possibility that the association of lymphocytes with the sulphide-containing phagocytes might result in the development of an immune response is also considered.

This work was supported partly by a Sir Henry Wellcome Travelling Fellowship, and partly by a grant from the National Cancer Institute of Canada. I should like to thank Dr. J. P. W. Gilman for his generous provision of facilities at his laboratory, and am also grateful to Dame Honor Fell, F.R.S. and Dr. A. Glücksmann, for their helpful comments on the manuscript. Some of the autopsies and histological preparations were made by Miss Winifred Smith ; the photographs were taken by Mr. M. Applin.

### REFERENCES

BERTHRONG, M. AND GRIFFITH, P.—(1961) *J. Path. Bact.*, **82**, 287.
FAGRAEUS, A.—(1948) *Acta med. scand.*, **130** (Suppl. 204), 1.
FIELD, E. J.—(1960) In 'Structure and Function of Muscle'. Edited by G. H. Bourne. New York (Academic Press) Vol. III, p. 148.
FISHMAN, M.—(1961) *J. exp. Med.*, **114**, 837.
GILMAN, J. P. W.—(1962) *Cancer Res.*, **22**, 158.
GILMAN, J. P. W. AND RUCKERBAUER, G. M.—(1962) *Cancer Res.*, **22**, 152.
HALPERN, B. N.—(1959) *J. Pharm. Pharmac.*, **11**, 321.
HARRIS, G.—(1966) *Nature, Lond.*, **211**, 154.
MASON, K. E.—(1960) In ' Structure and Function of Muscle '. Edited by G. H. Bourne. New York (Academic Press) Vol. III, p. 172.
PAYNE, W. W.—(1964) *Proc. Am. Ass. Cancer Res.*, **5**, abstr. 197.


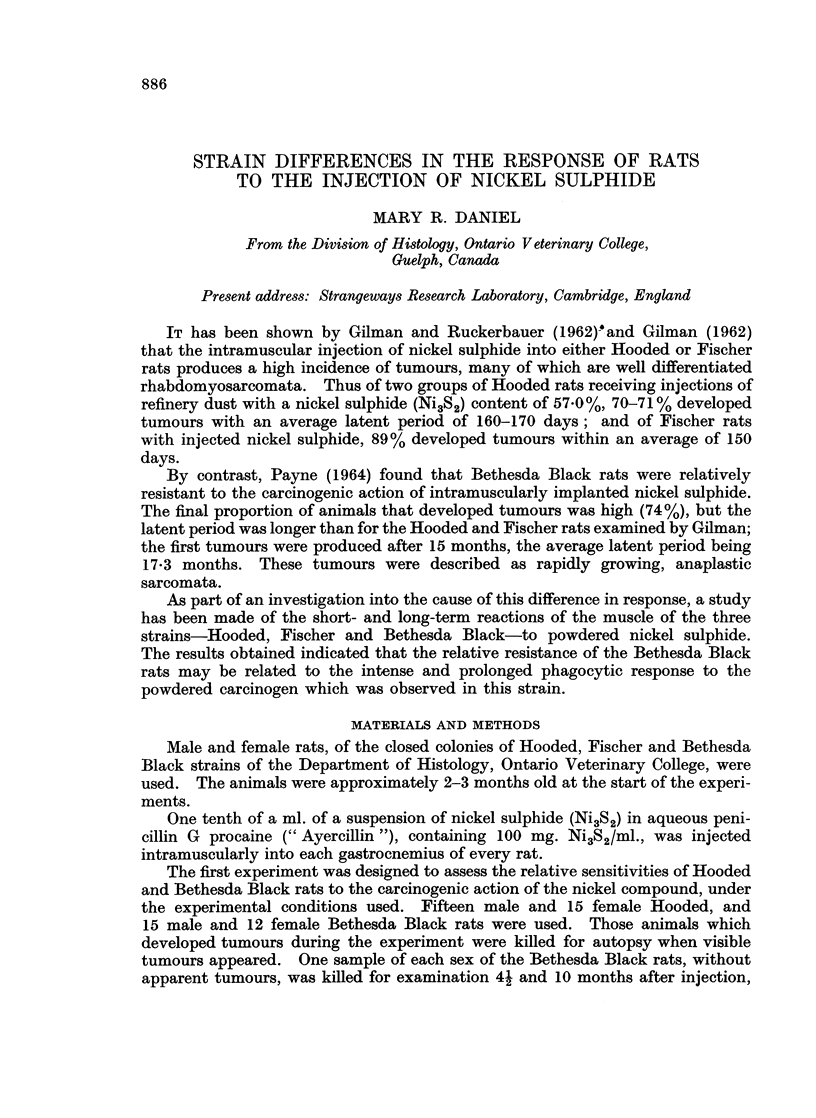

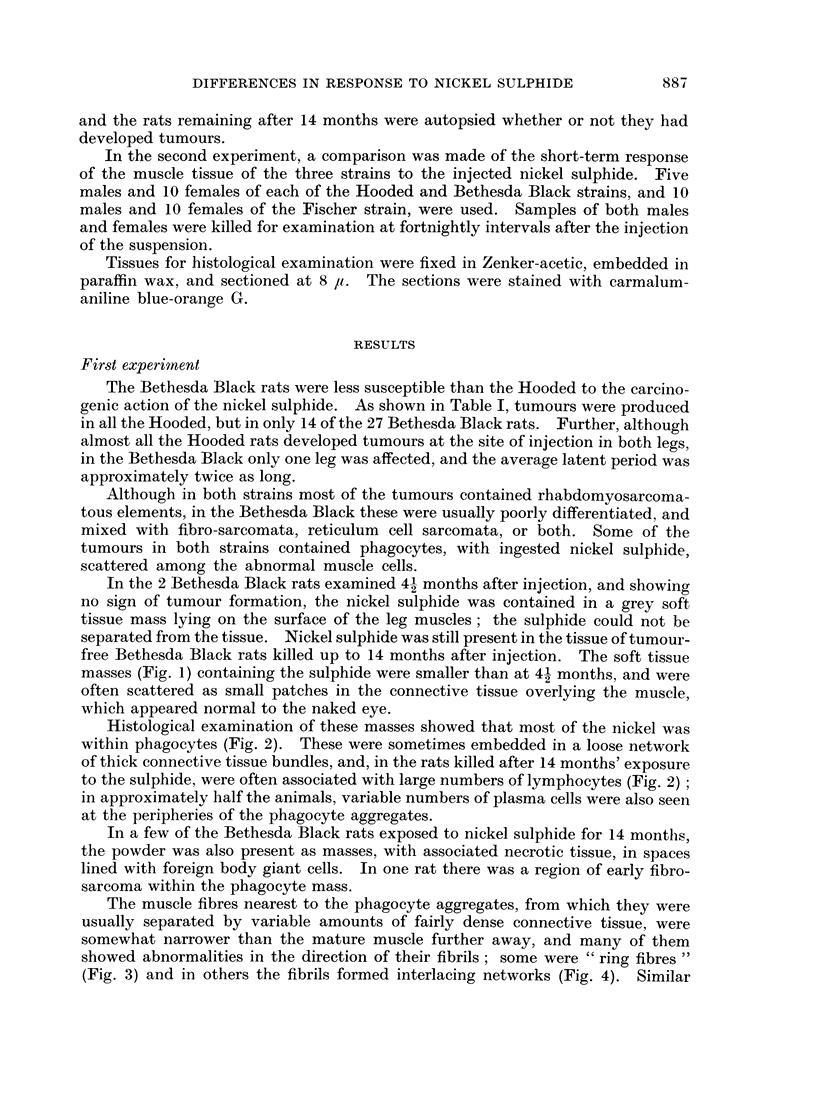

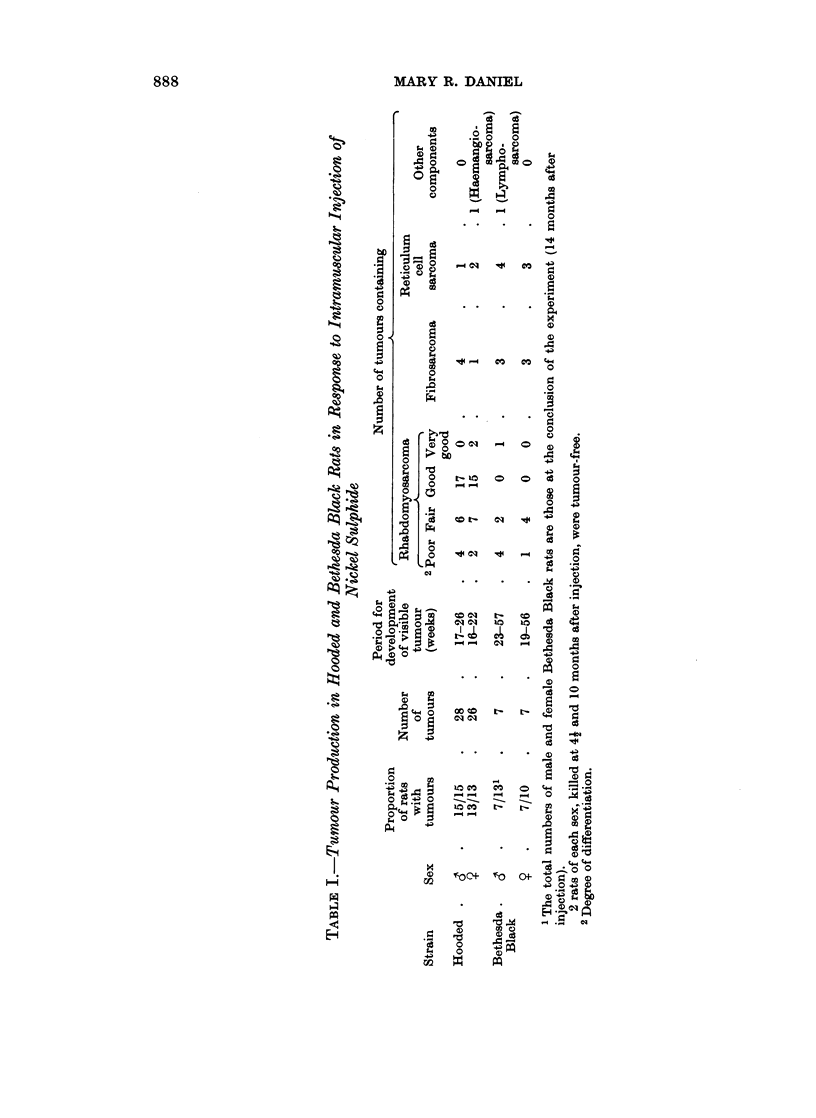

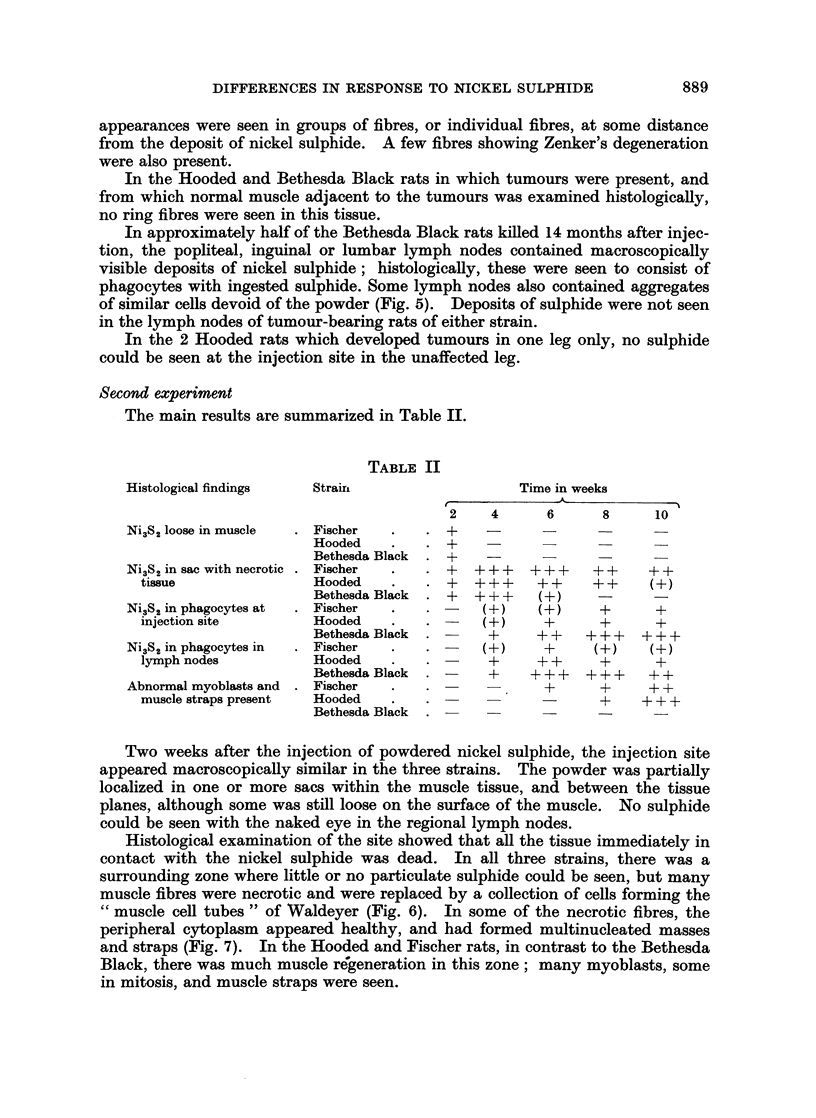

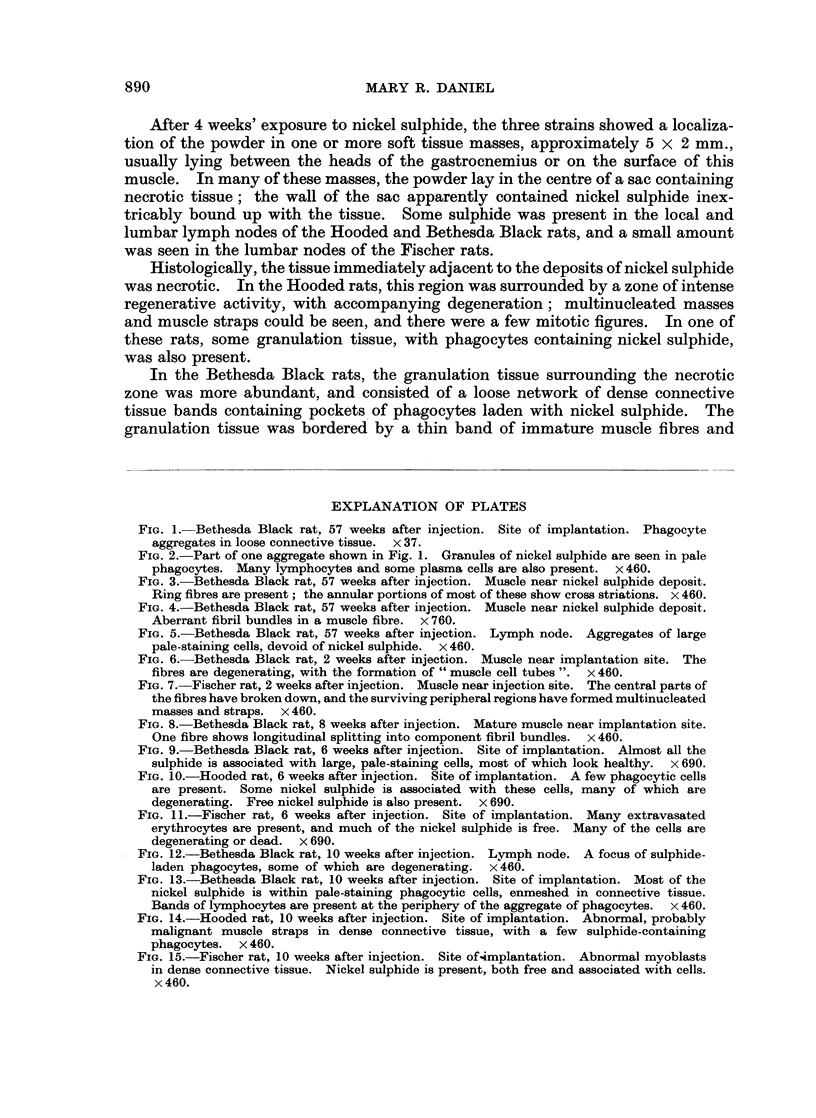

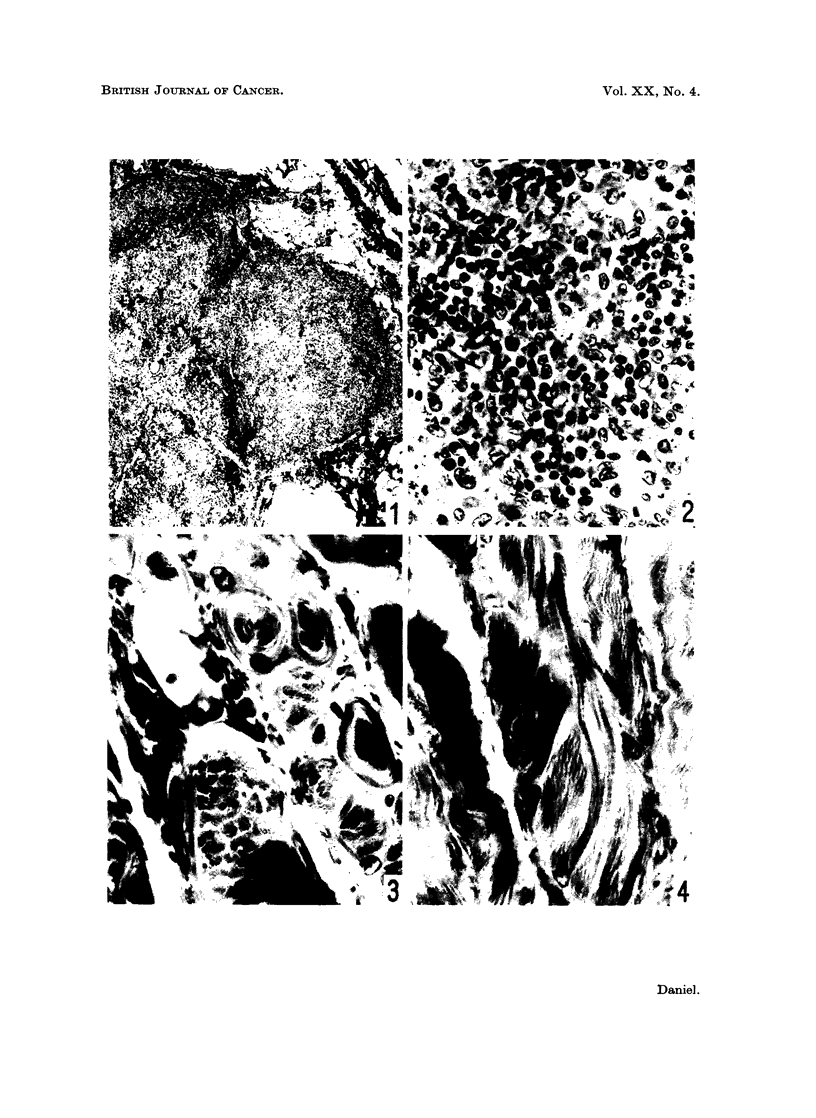

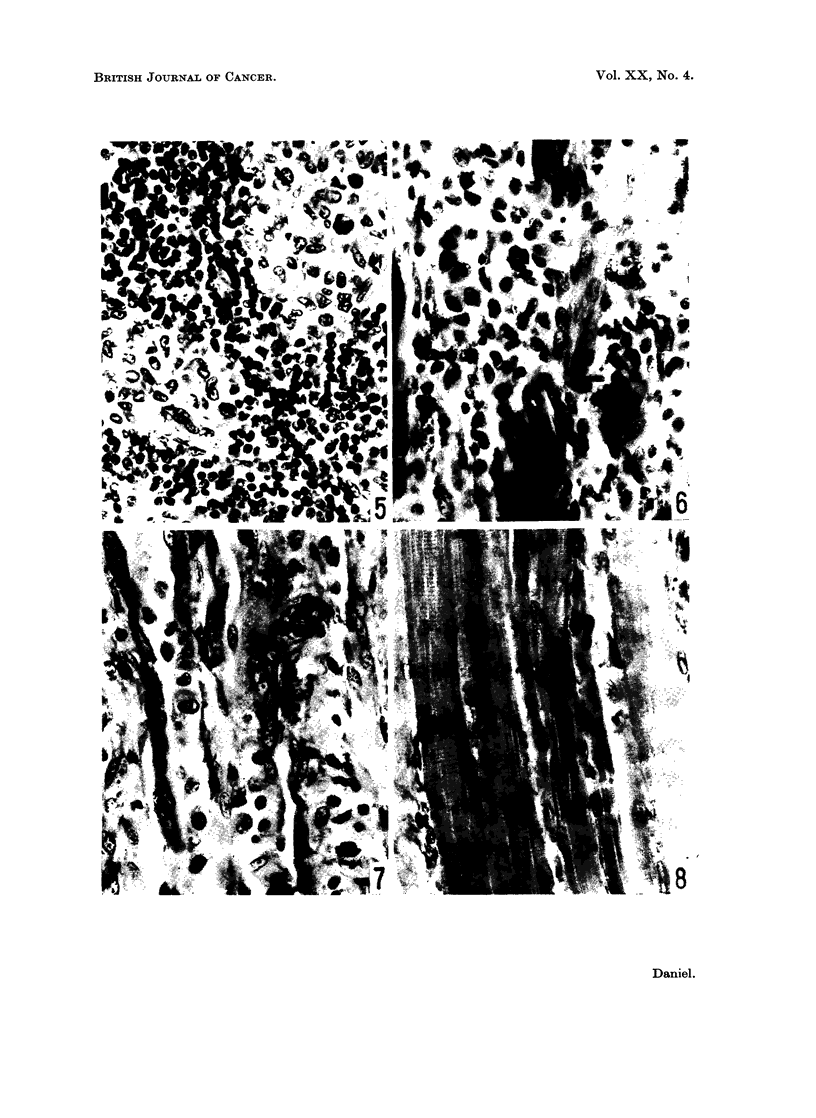

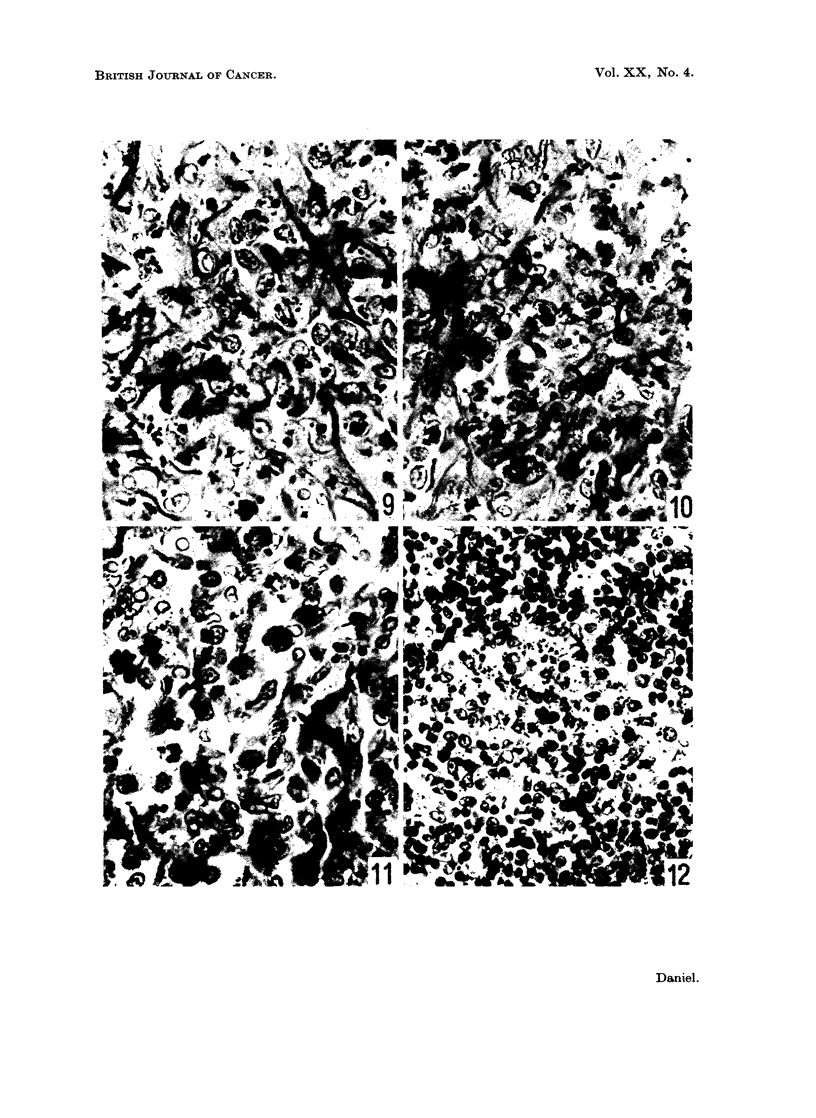

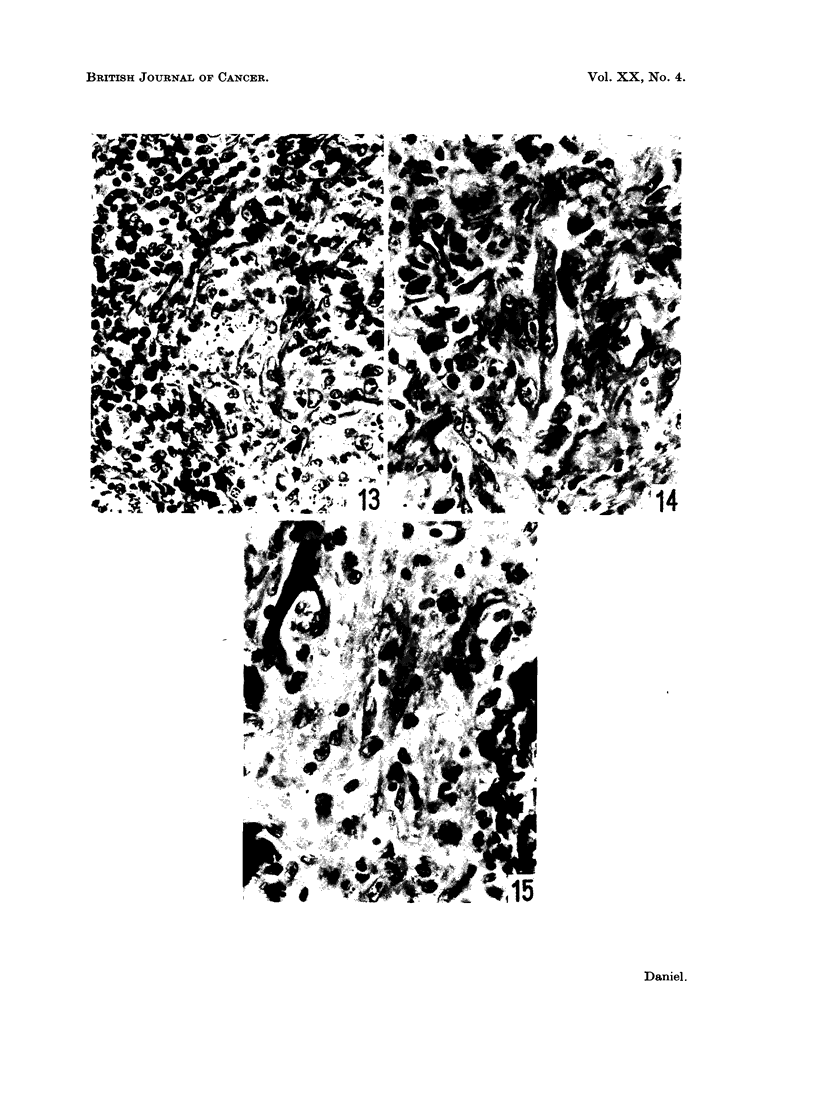

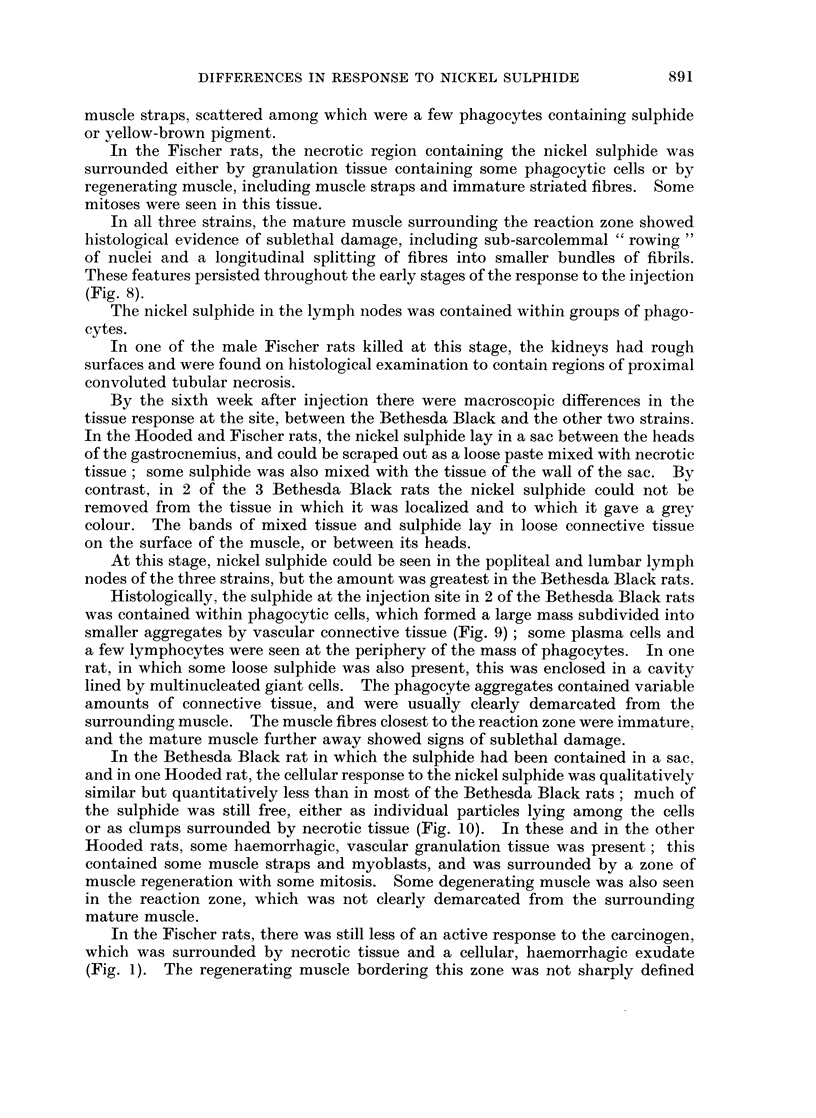

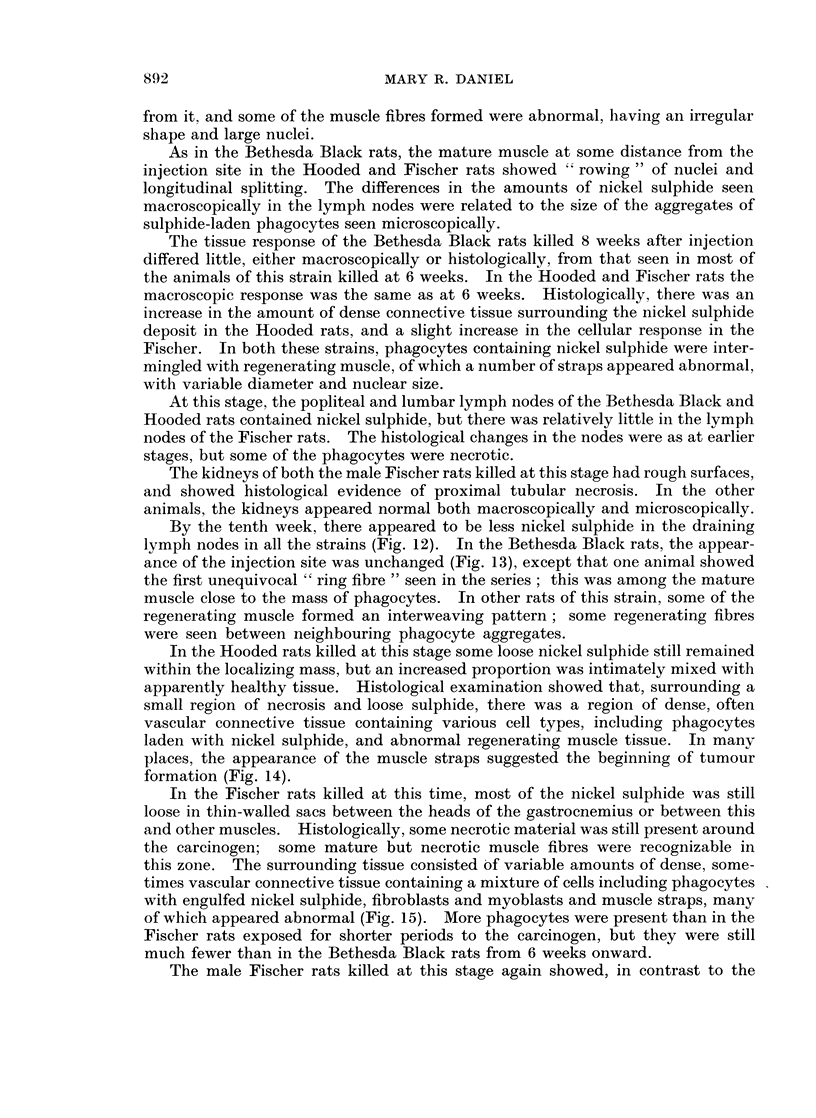

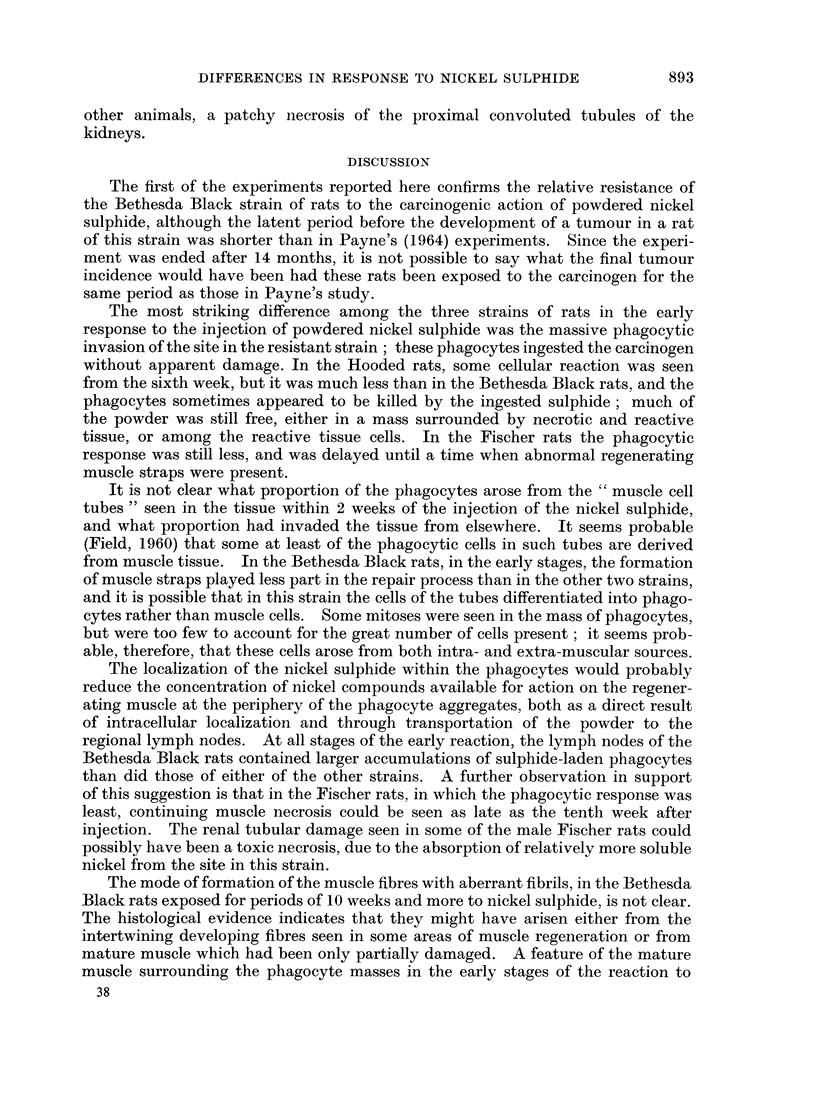

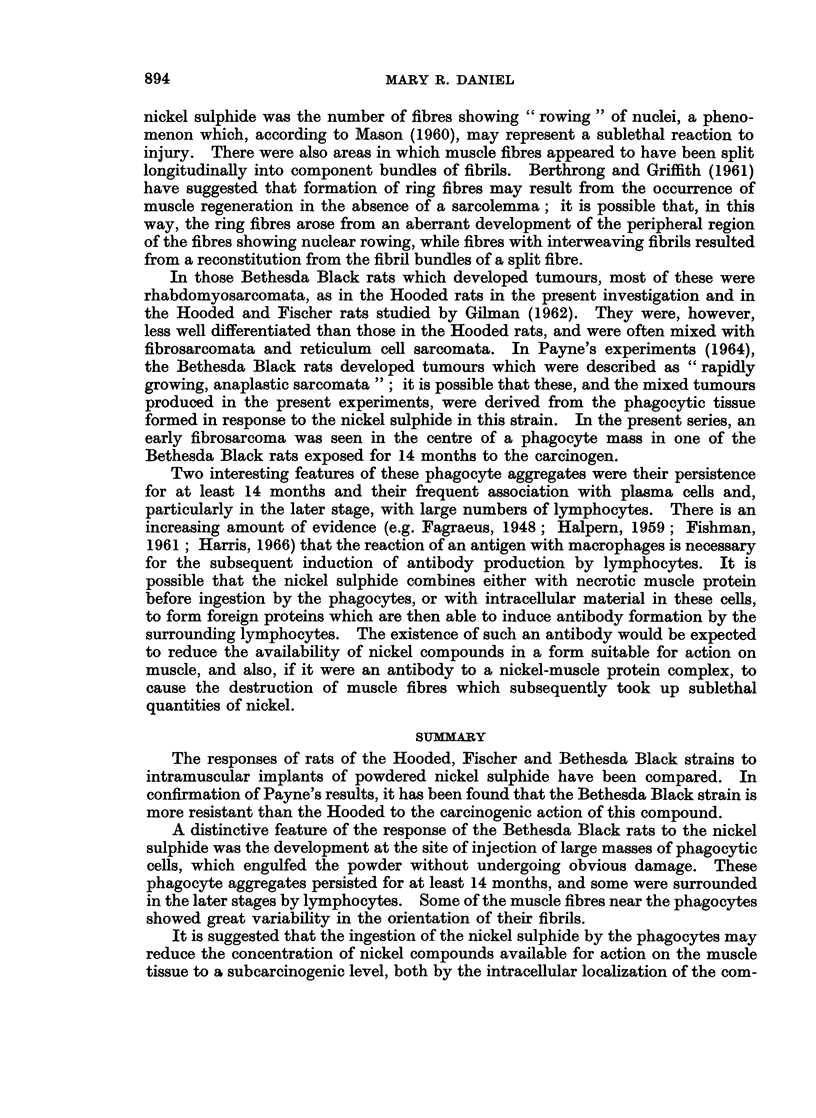

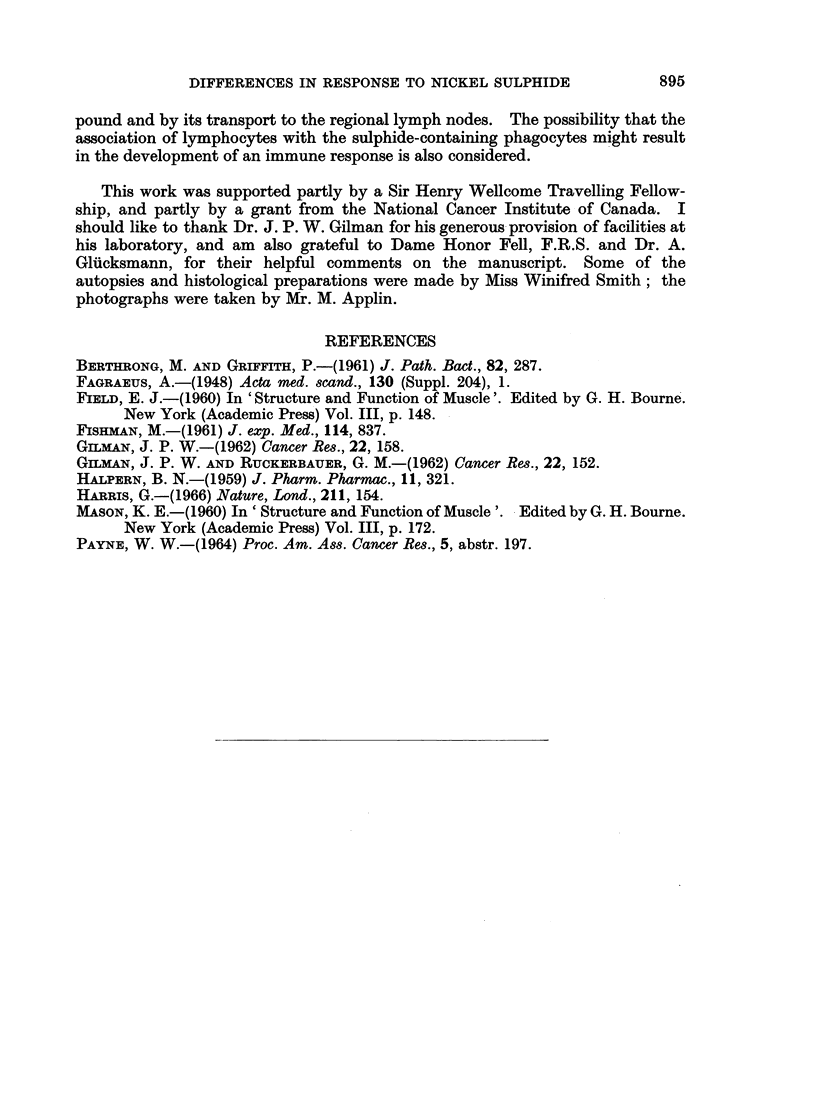


## References

[OCR_00844] BERTHRONG M., GRIFFITH P. (1961). Ring forms in skeletal muscle.. J Pathol Bacteriol.

[OCR_00850] FISHMAN M. (1961). Antibody formation in vitro.. J Exp Med.

[OCR_00852] GILMAN J. P. (1962). Metal carcinogenesis. II. A study on the carcinogenic activity of cobalt, copper, iron, and nickel compounds.. Cancer Res.

[OCR_00854] GILMAN J. P., RUCKERBAUER G. M. (1962). Metal carcinogenesis. I. Observations on the carcinogenicity of a refinery dust, cobalt oxide, and colloidal thorium dioxide.. Cancer Res.

[OCR_00855] HALPERN B. N. (1959). The role and function of the reticulo-endothelial system in immunological processes.. J Pharm Pharmacol.

[OCR_00856] Harris G. (1966). Ribonucleic acid synthesis in macrophages in relation to the secondary immune response in vitro.. Nature.

